# Mechanistic insights into heat shock protein 27, a potential therapeutic target for cardiovascular diseases

**DOI:** 10.3389/fcvm.2023.1195464

**Published:** 2023-05-12

**Authors:** Yifei Zou, Henghe Shi, Ning Liu, He Wang, Xianjing Song, Bin Liu

**Affiliations:** Department of Cardiology, The Second Hospital of Jilin University, Changchun, China

**Keywords:** heat shock protein 27, phosphorylation, cardiovascular diseases, cardiovascular pathophysiology, therapy

## Abstract

Heat shock protein 27 (HSP27) is a small chaperone protein that is overexpressed in a variety of cellular stress states. It is involved in regulating proteostasis and protecting cells from multiple sources of stress injury by stabilizing protein conformation and promoting the refolding of misfolded proteins. Previous studies have confirmed that HSP27 is involved in the development of cardiovascular diseases and plays an important regulatory role in this process. Herein, we comprehensively and systematically summarize the involvement of HSP27 and its phosphorylated form in pathophysiological processes, including oxidative stress, inflammatory responses, and apoptosis, and further explore the potential mechanisms and possible roles of HSP27 in the diagnosis and treatment of cardiovascular diseases. Targeting HSP27 is a promising future strategy for the treatment of cardiovascular diseases.

## 1. Introduction

According to the World Health Organization (WHO), cardiovascular diseases (CVDs) lead the world in terms of morbidity and mortality. By 2030, approximately 23.6 million people are predicted to die from these diseases, mainly stroke and heart disease (1). The cardiovascular system is highly energy-dependent, and homeostatic imbalances due to various causes can severely damage to the heart in a short period of time. Therefore, the cardiovascular system possesses a well-established set of protective mechanisms, including collateral circulation, the antioxidant system, and other intracellular stress responses that protect cardiovascular function. Among these defense schemes, the heat shock response (HSR) is a well-preserved evolutionary feature that induces the expression of heat shock proteins (HSPs) (2).

**Figure 1:**
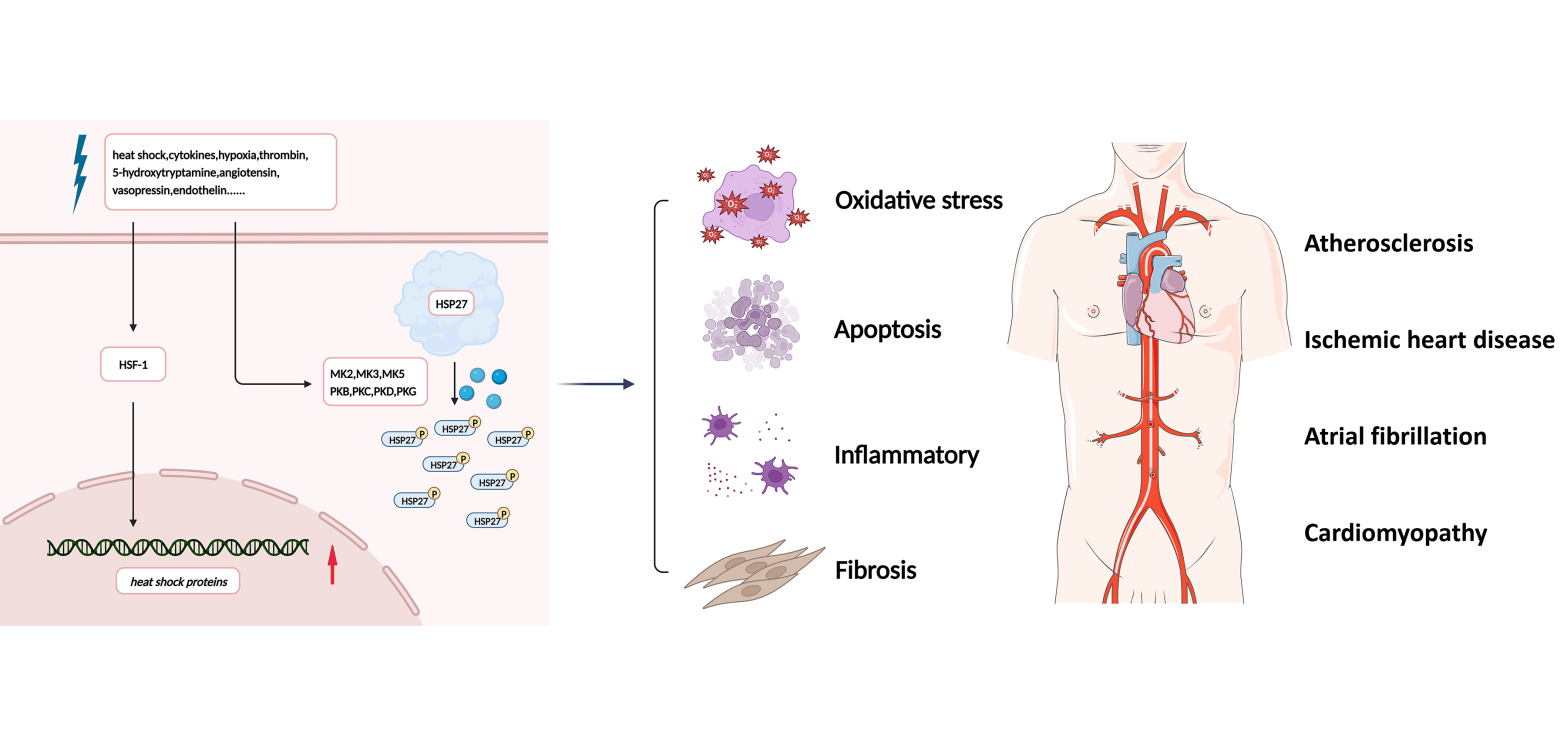
Simplified overview of the effects of HSP27. In the presence of stress stimuli such as heat shock, cytokines, hypoxia, thrombin, 5-hydroxytryptamine, angiotensin II, vasopressin, and endothelin, HSP27 is rapidly phosphorylated, leading to conformational changes and depolymerization, associated with signaling processes involving oxidative stress, inflammatory, apoptosis, and fibrosis. Future HSP27-related research in cardiovascular diseases remains to be conducted. HSF-1, heat shock transcription factor 1; HSP27, heat shock protein 27; MK2, MAPK activated protein kinase 2; PKB, protein kinase B; PKC, protein kinase C; PKD, protein kinase D; PKG, protein kinase G.

HSPs are a highly conserved family of proteins expressed by many cell types after exposure to stressful environmental conditions. The expression of HSPs increases with stresses such as hyperthermia, hypoxia, nutritional deficiencies, oxidative stress, and UV radiation, as well as under conditions associated with systemic stress, such as in response to ischemia-reperfusion injury (IRI) (3–5). Many HSPs have been shown to be molecular chaperones involved in the refolding of other damaged proteins. Small heat shock proteins (sHSPs) are dynamic polypeptides that are predominantly β-folded and form large oligomers. Some important functions that they exhibit include preventing protein aggregation, refolding proteins, conferring heat resistance to cells, and anti-apoptotic functions. sHSPs are the most stress-inducible molecular chaperones that prevent stress-induced aggregation of partially denatured proteins. They also exhibit refolding abilities similar to large HSPs but are not dependent on adenosine triphosphate (ATP) hydrolysis (6).

Heat shock protein 27 (HSP27) is a ubiquitous sHSP with chaperone activity. Previous studies have found that HSP27 is involved in and plays a protective role in CVD-related pathophysiological processes such as oxidative stress, inflammatory responses, and apoptosis (7). In this review, we first introduce the origin of HSP27 and the phosphorylation reactions closely related to its function, followed by a comprehensive and systematic summary of the pathophysiological mechanisms involved in the regulation of HSP27. We finally summarize and discuss the latest research advances in its use as a diagnostic and prognostic biomarker for CVDs. The prospects of targeting HSP27 for CVD treatment are promising.

## 2. Overview of HSP27

### 2.1. Heat shock proteins

The HSR is an evolutionarily highly conserved process that induces the expression of molecular chaperones in response to cellular stress and promotes the refolding of denatured proteins. As early as the 1930s, researchers have conducted experiments on the effects of heat stress on *Drosophila*, and the appearance of chromosome expansion after heat stress indicated enhanced local gene transcription (8). In addition to elevated temperatures, HSR can be activated by a variety of stressors, such as oxidative stress, glucose depletion, and overexpression of misfolded proteins (9). Heat shock transcription factor 1 (HSF1) activates the genes encoding HSPs when host cells are exposed to stress conditions such as heat shock, local ischemia, or toxic substances (10). This family of proteins, together with other chaperone proteins, prevents intracellular protein misfolding and aggregation, and promotes cellular repair after stress injury. Although they are known as heat shock or heat stress proteins, many HSPs are also commonly expressed in cells under physiological conditions and are essential for the maintenance of cellular homeostasis, such as proteostasis (11).

HSPs have molecular weights of 10–150 kDa. Mammalian HSPs can be divided into different families based on their molecular weights, including the chaperone protein families of sHSPs (HSP27), HSP40, HSP60, HSP70, HSP90, and large HSPs (HSP110 and GRP170) (12). In contrast to other major chaperone protein families, sHSPs bind and sequester non-natural and denatured proteins in an ATP-independent manner, facilitating subsequent substrate depolymerization and/or refolding (13). sHSPs are characterized by low molecular weights (12–43 kDa, average length of 160 residues) and a conserved alpha-crystallin protein structural domain (alpha-crystallin domain, ACD). The highly conserved ACD consists of 90–100 amino acids and forms an immunoglobulin-like β-sandwich structural domain with seven to eight antiparallel β-sheet layers. The ACDs of human sHSPs are rich in histidine, which improves the ability to respond to changes in pH and metal ion effectiveness and regulates sHSP activity. ACDs are flanked by a highly variable amino-terminal region (NTR) and a flexible carboxy-terminal region (CTR). The NTR is rich in hydrophobic residues and is highly disordered, while the CTR is rich in charged polar residues, which allows extremely high concentrations of sHSPs to remain soluble in the cell. Both the NTRs and CTRs exhibit high flexibility and wide sequence variation, and contribute to the oligomerization of sHSPs (14), a process that will be elaborated in the next section.

### 2.2. HSP27 and its phosphorylation

HSP27 (also known as HSPB1) is a member of the sHSP family. In addition to the conserved ACD region, the amino-terminal WDPF structural domain of HSP27 is essential for oligomerization, and the carboxy-terminal flexible variable region is thought to be involved in interactions with target proteins, regulation of solubility, and oligomerization. The presence of 10.2% serine, 6.8% threonine, and 2.4% tyrosine residues in the primary sequence of HSP27 provides a structural basis for HSP27 phosphorylation. The amino-terminal Ser15, Ser78, and Ser82 are now known to be important phosphorylation acceptor sites (15). HSP27 phosphorylation regulates its oligomerization. Typically, unphosphorylated HSP27 exists as large multimers that can oligomerize into large aggregates of up to 800 kDa or form heterodimeric structures with other members of the sHSP family. In the presence of stress stimuli such as heat shock, cytokines, hypoxia, thrombin, 5-hydroxytryptamine, angiotensin II, vasopressin, and endothelin, HSP27 is rapidly phosphorylated, leading to conformational changes and depolymerization (16). In cells, HSP27 phosphorylation is required for most of its functional activities (17). Phosphorylated HSP27 exists mainly as a monomer to a tetramer and acts as an inhibitor of apoptosis and an inducer of autophagy (18).

Under physiological conditions, HSP27 is constitutively expressed in a variety of cells and is upregulated under stress conditions, helping to maintain intracellular protein conformations and stabilize protein functions. HSP27 is also associated with signaling processes involving cell proliferation, cell cycle regulation, apoptosis, autophagy, and fibrosis (19–21). In recent years, clinical studies have identified HSP27 as a potential target and biological marker for the diagnosis, treatment, and prognosis of a variety of diseases, including cancer (15, 22, 23), neurological diseases (24–26), and CVDs (27–29). In the following section, the role of HSP27 and its phosphorylation in the regulation of pathophysiological processes, such as oxidative stress and inflammatory responses, will be discussed and analyzed in a comprehensive and systematic manner (Figure 1).

## 3. Pathophysiological processes regulated by HSP27

### 3.1. Oxidative stress

In recent years, HSP27 has been extensively studied in redox biology. HSP27 has been shown to regulate intracellular redox homeostasis and enhance cellular resistance to oxidative damage by protecting against lipid peroxidation (LPO) and protein oxidation (30).

#### 3.1.1. Oxidative stress and HSP27 expression levels

In the cardiovascular system, protein quality control system (PQS) disorders are critical for the progression of hypertrophic cardiomyopathy (HCM) (31). Elevated HSP27 expression and its hyperphosphorylation are present in HCM hearts. Exogenous supplementation with HSP27 reduces oxidative stress levels in HCM myocytes, ameliorates PQS disorders, and reduces myocardial stiffness (32). *In vitro* experiments revealed that HSP27 prevents oxidative stress by increasing glucose-6-phosphate dehydrogenase (G6PD) activity and maintaining reduced glutathione (GSH) levels (33). After administration of ethanol and methylenedioxymethamphetamine to adolescent mice, sympathetic pathways were activated and cellular stress was increased. Increased HSP27 in the right ventricle may increase thioredoxin-1 (Trx1) levels and reduce the oxidative stress induced by the co-stimulation with both drugs (34).

HSP27 overexpression significantly ameliorated oxidative stress. Upregulation of HSP27 improved the reduced state of glutathione reductase (GR), peroxiredoxin-1 (Prx1), and Trx1 in H9c2 cells treated with hydrogen peroxide, increased the GSH/ glutathione disulfide (GSSG) ratio, promoted the formation of a complex between HSP27 and oxidized Prx1, regulated the redox status of key proteins involved in the cytosolic antioxidant pathway, and protected H9c2 cells from oxidative stress (35). In addition, HSP27 regulated the Hippo pathway by enhancing dephosphorylation of mammalian STE20-like kinase 1, leading to reduced phosphorylation of large tumor suppressor kinase 1 (LATS1) and Yes-associated protein (YAP) and increased nuclear localization of YAP, which ameliorated the hydrogen peroxide-induced imbalance in the cytoplasmic redox state and drove the transcription of genes associated with cytoprotection (35). Drosophila larvae exposed to nonylphenol exhibited significantly reduced midgut HSP27 and ecdysone receptor mRNA levels, decreased GSH levels and thioredoxin reductase activity, and increased levels of reactive oxygen species (ROS). This was accompanied by a nonylphenol concentration-dependent increase in LPO, protein carbonyl (PC) content, and cell death. Overexpression of HSP27 ameliorated oxidative stress by decreasing nonylphenol-induced intracellular ROS, LPO, and PC levels in the midgut, and decreased GSH levels (36). Cyclo(His-Pro) increased soluble superoxide dismutase (SOD) activity by upregulating HSP70 and HSP27 and decreasing lipopolysaccharide (LPS)-induced ROS production (37).

Altering HSP27 cross-linking altered normal HSP27 dimerization, inhibited HSP27 function. HSP27 inhibitors downregulated radiation-induced ROS production, inhibited immune cell infiltration in lung tissue (38). HSP27 knockdown decreased human dermal fibroblast viability and increased ultraviolet B (UVB)-induced ROS production. Compared to UVB exposure alone, HSP27 knockdown in UVB-exposed cells resulted in significantly lower LC3B expression, significantly higher p62 expression, and reduced expression of some antioxidants, such as SOD and catalase, accelerating UVB-induced ROS release (39). The use of intermittent pressure-imitating, rolling manipulation to simulate traditional Chinese medicine manipulation in a human skeletal muscle cell line increased SOD activity and decrease malondialdehyde (MDA) content and creatine kinase activity by downregulating CD36, HSP27, and FABP4 expression. This improved oxidative stress and lipid metabolic imbalances (40).

#### 3.1.2. Oxidative stress and HSP27 phosphorylation

There is also a degree of modulation between HSP27 phosphorylation levels and oxidative stress signaling pathways. As a stress response, freezing stimulation can lead to enhanced p38 kinase activity and increased HSP27 phosphorylation, which inhibits oxidative stress and exerts a protective effect by increasing cytoplasmic SOD1 levels (41). Yusuke et al. found (42) that during cerebral ischemia/reperfusion (I/R), ataxia telangiectasia mutated (ATM) kinase, via phosphorylation of HSP27 on serine 85, induced G6PD activity in the pentose phosphate pathway to increase the NADPH/NAD+ ratio and exert antioxidant effects. ATM inhibitors decreased HSP27 phosphorylation and G6PD upregulation after I/R, significantly increasing PC levels and leading to an increased infarct size. Geranylgeranylacetone did not affect HSP27 levels in brain I/R-injured rats but significantly increased HSP27 phosphorylation and reduced PC levels by increasing G6PD activity (43). However, the exact mechanism needs to be confirmed in future studies.

These results indicate that HSP27 expression and its phosphorylation regulate many enzymes involved in oxidative stress, thus reducing ROS production. However, the specific mechanism by which HSP27 regulates enzymes and how it affects their expression levels are unclear and need to be determined by more research.

### 3.2. Inflammatory responses

HSP27 is a major intracellular molecular chaperone and a regulator of inflammatory signaling responses. Exogenous HSP27 prevents neutrophil apoptosis in a dose-dependent manner without altering the levels of inflammatory cytokines such as interleukin (IL) 12 and IL-10, prolonging neutrophil survival and exacerbating acute inflammation and tissue destruction (44). The combination of inflammatory markers, soluble suppression of tumorigenicity 2 (sST2), HSP27, and high-sensitivity C-reactive protein is an important factor in patients with heart failure (HF) as an independent predictor of cardiovascular death and unplanned HF-related hospitalization (45). A positive correlation can be found between systemic HSP27 levels and the pro-inflammatory cytokines tumor necrosis factor alpha (TNF-α) and IL-6 (*p* < 0.001) (46).

#### 3.2.1. Inflammatory responses and HSP27 expression

Upregulation of intracellular HSP27 expression inhibits the inflammatory response. *Clostridium difficile* (CD) causes severe gastrointestinal inflammatory diarrhea in patients treated with antibiotics. It was found that overexpression of HSP27 protected the levels of cystic fibrosis transmembrane regulator (CFTR), Na^+^/H^+^ exchange regulatory factor-1 (NHERF1), and Na^+^/H^+^ exchange regulatory factor-2 (NHERF2), rendering them unaffected by toxin CD B (TcdB). Thus, the binding of CFTR to NHERF1 and NHERF2 was reduced, preventing a decrease in transepithelial resistance, and protecting intestinal barrier function (47). Upregulation of HSP27 is involved in the regulation of cellular resistance to *Escherichia coli* F18 by inhibiting adhesion of the bacteria to porcine small intestinal epithelial cells and reducing pro-inflammatory factor concentrations that drive exogenous stress-induced inflammatory responses. In addition, upregulation of *HSP27* gene expression led to upregulation of the *ribosomal S6 kinase 2* (*RSK2*) gene in the mitogen-activated protein kinase (MAPK) pathway, which synergistically counteracted stress-induced inflammatory responses in the body (48). Valdecoxib attenuated nuclear factor-κB (NF-κB) and IκB phosphorylation in mouse skeletal muscle by increasing 5′-adenosine monophosphate-activated protein kinase (AMPK) phosphorylation and HSP27 expression via the AMPK pathway. Valdecoxib also reduced eukaryotic initiation factor 2 alpha phosphorylation and C/EBP homologous protein (CHOP) expression, decreased serum levels of the pro-inflammatory cytokines TNF-α and IL-6, and improved the inflammatory response and endoplasmic reticulum (ER) stress (49). In rats (50), renal IRI increased serum creatinine and urea nitrogen concentrations; promoted LPO by elevating MDA levels; inhibited SOD activity; induced NF-κB, IL-2, and TLR-4 expression to stimulate inflammatory responses; and increased renal cell apoptosis. Hydrogen sulfide protected the renal tissue from LPO, inflammation, and apoptosis, possibly due to the upregulation of HSP70, heme oxygenase 1, and HSP27. HSPB1 knockout mice subjected to myocardial infarction (MI) exhibited enhanced and prolonged leukocyte infiltration, enhanced inflammatory cytokine expression, and enhanced toll-like receptor 4 (TLR4)/myeloid differentiation factor 88 (MyD88)/NF-κB activation in their heart tissue. Further analysis of primary mouse cardiomyocytes showed that cardiomyocyte-specific HSPB1 knockdown increased NF-κB activation, promoted the expression of proinflammatory mediators, increased leukocyte recruitment, and activated excessive inflammatory responses, ultimately leading to poor remodeling, cardiac dysfunction, and cardiac rupture after MI (51). HSP27 inhibited the expression of transcriptional repressor E2F4/p130 to promote cell cycle progression. HSP27 knockdown resulted in increased expression of E2F4/p130 and downregulation of the expression of six G2/M-related genes (CCNA2, CCNB1, CCNB2, CDC25C, CDCA3, and CDK1), leading to G2 blockade and upregulation of inflammatory factors and cellular senescence (52).

#### 3.2.2. Inflammatory responses and HSP27 phosphorylation

Inflammatory mediators trigger the inflammatory response through G protein-coupled receptors (GPCRs), including protease-activated receptor 1 (PAR1). Thrombin ligand activation of PAR1 stimulates pro-inflammatory p38 MAPK signaling and promotes endothelial barrier disruption. GPCRs induce the p38-dependent MAPK-activated protein kinase 2 (MAPKAPK2)-MAPKAPK3 inflammatory signaling pathway that mediates HSP27 phosphorylation and activation to control endothelial barrier restoration and vascular leakage. Knockdown of HSP27 or blockade of HSP27 oligomer formation enhances endothelial barrier permeability *in vitro* and vascular leakage *in vivo* in response to PAR1 activation (53). Noxa inhibits the nuclear translocation and activation of NF-κB by inhibiting the degradation of ubiquitinated IκBα through the interaction of covalent disulfide bonds with phosphorylated HSP27, thereby suppressing the inflammatory response to allergens (54).

HSP27 phosphorylation promotes CREB-binding protein (CBP) transcriptional output via p53 activation during early inflammation in LPS-stimulated THP-1 cells, regulates NF-κB activation, and increases the release of the inflammatory factors TNF-α, IL-6, interferon-beta (IFN-β), and IL-10. In late inflammation, phosphorylated HSP27 inhibits an excessive increase in CBP by suppressing the excessive accumulation of ROS. The phosphorylated state of HSP27 controls the LPS-induced inflammatory response by regulating CBP (55). HSP27 was phosphorylated at serine 78 and 82 after exposure to LPS. Inhibition of HSP27 phosphorylation significantly exacerbated LPS-induced apoptosis, growth inhibition, and inflammatory factor expression by inhibiting IκBα phosphorylation and NF-κB activation (56). High mobility group box-1 protein (HMGB1) is a pro-inflammatory cytokine associated with death during sepsis and other inflammatory diseases. After acetylation by CBP at transcriptional junctions, HMGB1 is translocated from the nucleus to the cytoplasm. In the cytoplasm of static monocytes, HSP27, in a non-phosphorylated state, interacts with CBP as a natural regulator to promote ubiquitin-mediated CBP degradation. After LPS stimulation, HSP27 is phosphorylated through the p38 MAPK/MK2 signaling pathway, translocates to the nucleus to bind CBP, inhibits CBP acetyltransferase activity and subsequent CBP-dependent acetylation of HMGB1, prevents HMGB1 translocation to the cytoplasm, and inhibits inflammatory signaling (57).

#### 3.2.3. Inflammatory responses and HSP27 immune complex

Blood levels of HSP27 and natural IgG autoantibodies (anti-HSP27 antibodies, AAb) were higher in healthy controls than in patients with CVD (58). The interaction of the HSP27 immune complex (IC) with TLR4 was found to enhance binding to the membrane of THP-1 macrophages (MΦ), activate the NF-κB pathway, and competitively displace the interaction of LPS with this receptor. The activation of NF-κB via this external pathway affects the anti-inflammatory milieu, with increased secreted IL-10 and decreased IL-1β levels. In addition, HSP27 IC competes with oxidized low-density lipoprotein (oxLDL) to bind the scavenger receptors SR-AI and CD-36, reducing oxLDL internalization and foam cell formation. This finding suggests that HSP27 IC can act as a natural antagonist of inflammatory signaling and MΦ foam cell formation (59). Further studies on THP-1 MΦ showed (60) that HSP27 IC increased the number of secreted exosomes and the concentration of cholesterol in exosomes. HSP27 immunotherapy is not only a preventive measure for atherosclerosis but also a potential treatment for atherosclerosis by reducing the cholesterol content of plaques that have already formed.

#### 3.2.4. Inflammatory responses and exosomal HSP27

In addition to intracellular chaperone functions, cellular stress proteins can be released by cells and are present in extracellular environments, such as in exosomes or body fluids, and are increasingly used as biomarkers of human disease states (61). Shi et al. (62) found that HSP27 is present on exosomal membranes and that exosomes containing HSP27, as an important exosomal cargo exerting anti-inflammatory effects, stimulate NF-κB activation and IL-10 release. This finding suggests that the future use of exosomes to target specific tissues and organs for the delivery of protein cargoes for precision therapy is an interesting possibility.

### 3.3. Apoptosis

It is well known that HSP27 is involved in endogenous and exogenous apoptotic pathways and interacts with apoptotic mediators to exert anti-apoptotic effects.

#### 3.3.1. Apoptosis and HSP27 expression levels

HSP27 plays an active antiapoptotic role in the pathophysiology of several diseases. HSP27, Bax, and caspase-3 expression was upregulated in AS mouse plaques and ROS levels were elevated in the aorta. HSP27 knockdown resulted in increased plaque size, increased apoptosis, and enhanced aortic ROS levels. HSP27 maintained oxidative stress homeostasis and mitigated progression of atherosclerosis by inhibiting the mitochondrial apoptosis pathway (63). In a mouse I/R injury model (64), miR-410 inhibited mitochondrial autophagy after cardiac I/R injury by directly targeting HMGB1 to regulate HSP27 activity. Inhibition of HSP27 significantly attenuated the promoting effect of HMGB1 upregulation on cell viability and ATP production, as well as its inhibitory effect on apoptosis. Zhou et al. (65) found that HSP27 expression was elevated in rat neurons after lumbosacral nerve root avulsion injury. In a oxygen-glucose deprivation (OGD) model, upregulation of HSP27 was found to increase GSH content and SOD activity and to inhibit oxidative stress, suppressing OGD-induced apoptosis. HSP27 knockdown significantly reduced p65 phosphorylation and eliminated OGD-induced NF-κB activation. HSP27 may inhibit OGD-induced apoptosis by activating the NF-κB signaling pathway.

In a rat subarachnoid hemorrhage (SAH) model (66), HSP27 overexpression attenuated neurological defects and apoptosis in the basal cortex and effectively inhibited activation of MAP kinase kinase 4, c-Jun N-terminal kinase, c-Jun, and caspase-3, exerting neuroprotective effects. UVB irradiation triggered changes in HSP27 phosphorylation and localization, inducing the translocation of HSP27 from the cytoplasm to the nucleus and protecting the cells from UVB damage (67). HSP27 knockdown was found to increase levels of a chronic product of photoaging, MDA; decrease hydroxyproline content; upregulate protein expression of the aging markers p16, p53, and p21; and promote the expression of the pro-apoptotic factor Bax. These changes resulted in a significant decrease in collagen fibers and thickening and distortion of elastic fibers (68). Further studies have revealed that HSP27 regulates the subcellular localization of p21 by activating the phosphorylated protein kinase B (AKT)-dependent pathway and inhibiting the p53/Bax/Bcl-2-dependent mitochondrial apoptotic pathway to promote the transfer of p21 to the cytoplasm (69). Semisynthetic penicillinase-resistant antibiotics (PRAs) induce ER stress in hepatocytes, resulting in pure cholestasis. HSP27 activates the phosphatidylinositol 3-kinase (PI3K)/AKT pathway, which inhibits caspase-3 activity and prevents ER stress-triggered apoptosis (70). However, intravitreal injection of HSP27 also caused elevated levels of caspase-3, -8, and -9 in retinal cells, activation of intrinsic and extrinsic apoptotic pathways, and increased apoptosis, leading to retinal damage (71). The mechanism by which exogenous injection of HSP27 increases apoptosis remains to be investigated.

Since HSP27 is able to inhibit apoptosis and autophagy, this biological function can then be exploited by reducing HSP27 expression to promote tumor cell apoptosis. Resveratrol combined with small interfering RNA (siRNA)-mediated silencing of HSP27 promotes caspase-3-induced apoptosis (72). Widespread chemotherapy resistance can lead to a decrease in the effectiveness of anticancer drug therapy. Inhibition of HSP27 decreases NOTCH1 expression and the phosphorylation of AKT and mechanistic target of rapamycin (mTOR), enhances the effects of 5-fluorouracil (5-FU) and vincristine on the downstream AKT-mTOR pathway, and increases chemotherapeutic drug-related apoptosis (73). Both apoptosis and autophagy were strongly induced curcumin treatment, resulting in the effective treatment. Knockdown of HSP27 increased the resistance of cells to curcumin through the p-AKT/AKT signaling pathway; decreased reactive oxygen/nitrogen species, superoxide, and autophagy levels and reduced reactive oxidative stress and apoptosis (74). Radiation therapy is the treatment of choice in patients with limited prostate cancer (PCa). MiR-541–3p inhibited the expression of β-catenin by targeting HSP27 mRNA and its 3′ untranslated region (3′UTR), upregulated cell apoptosis after radiation (75). Heat stress was shown to downregulate miR-541 levels, leading to increased translation of HSP27. This promoted autophagy in normal lung bronchial epithelial cells through ATG7, increased mitochondrial membrane potential, reduced cytochrome C release from mitochondria into the cytoplasm (thereby inhibiting apoptosis), and acted as an oncogene in the heat stress-induced transformation of lung epithelial cells (76).Silencing HSPB1 increased the radiosensitivity by decreasing cell viability, depolarizing the mitochondrial membrane potential, arresting the cell cycle in the G2/M phase, and promoting apoptosis (77).

The promotion of HSP27 expression can play a positive cytoprotective role and reduce the occurrence of adverse drug reactions. Overexpression of HSP27 prevented apoptosis and maintained axonal integrity, leading to reversal of aberrant pain responses in paclitaxel-treated transgenic mice overexpressing human HSP27 (hHsp27 Tg mice). HSP27 overexpression exerted its anti-apoptotic function by inactivating caspase-3 and maintained axonal integrity by inactivating RhoA and its downstream effectors to prevent peripheral neuropathy (78). Vincristine-induced mechanical and cold abnormal pain was also prevented in the hHsp27 Tg mice by blocking axonal degeneration, demyelination, mitochondrial dysfunction, and apoptosis (79). HSP27 protected cardiomyocytes against doxorubicin-induced toxicity by upregulating the cytoprotective cascade system, specifically by upregulating enzyme activity or activating PI3K/AKT to attenuate the p53 cascade (80).

#### 3.3.2. Apoptosis and HSP27 phosphorylation

Phosphorylation of overexpressed HSP27 at Ser82 and its association with p53 are critical for cardioprotection in doxorubicin -induced dilated cardiomyopathy. Only phosphorylated HSP27 could protect the heart by inhibiting the p53-dependent apoptotic pathway (81). Glucocorticoids reduced TNF-α-induced apoptosis in human aortic smooth muscle cells and maintained aortic wall homeostasis by increasing the levels of unbound soluble tumor necrosis factor receptor-2 (TNFR2) and inhibiting phosphorylation of HSP27 induced by activation of the p38 MAPK-HSP27 pathway (82). Endolipin exerted a protective effect against high glucose-induced apoptosis in human umbilical vein endothelial cells (HUVECs) by promoting HSP27 phosphorylation through PI3K/AKT and ERK1/2 signaling pathways, and this protective effect could be significantly blocked by siRNA-HSP27 (83). Tanshinone IIA (TIIA) induces phosphorylation of HSP27 at serine 82, leading to activation of the unfolded protein response and accumulation of ROS, which induces the expression of HSF1 and its target genes and leads to cell apoptosis (84). The chemotherapy regimen FIRINOX (a combination of 5-fluorouracil, irinotecan, and oxaliplatin) leads to pancreatic ductal adenocarcinoma (PDAC)-induced DNA damage, activating the NF-κB pathway and TNF-α production. The resultant autocrine TNFR1 activity triggers apoptotic signaling and mediates the treatment-resistant TAK1-p38-MK2-HSP27 pathway. Targeting kinases that directly phosphorylate HSP27, thus blocking HSP27 phosphorylation, can promote apoptosis (85). Studies have found (86) that dioscin promotes ROS-induced apoptosis via the p38 MAPK/HSP27 pathway and that inhibition of HSP27 phosphorylation attenuates dioscin-mediated apoptosis. Therapeutic regimens targeting HSP27 and its phosphorylation can play an active role by regulating the apoptotic process.

### 3.4. Fibrosis

Fibrosis is the end stage of persistent tissue damage and chronic inflammatory responses, characterized by excessive accumulation of extracellular matrix and destruction of normal tissue structure. HSP27 has been shown to be overexpressed in the pleura of patients with pulmonary fibrosis and other fibrotic diseases (87). In a study of two mouse models of thrombopoietin and constitutively active JAK2 mutant (JAKV617F)-induced myelofibrosis (88), HSP27 was found to regulate the proliferation of JAK2V617F-positive cells and interact directly with JAK2/STAT5 to protect STAT5 from dephosphorylation. Thus, HSP27 may serve as a potential therapeutic target. In transforming growth factor beta (TGF-β)-stimulated human renal proximal tubule HK-11 cells and in the kidneys of diabetic mice (89), researchers demonstrated that pSer82 HSP27 can bind to nuclear factor erythroid 2 (NF-E2), which may promote NF-E2 degradation by targeting the protein to the proteasome, thereby promoting fibrosis. Recombinant HSP27 can be combined with antibodies targeting the angiotensin II type 1 (AT1) receptor to form a ZZ-TAT-GFP fusion protein that specifically targets cardiomyocytes, and can reduce apoptosis, reduce the area of myocardial fibrosis, increase ejection fraction, reduce end-systolic volume and end-diastolic pressure, and improve cardiac function after MI (90).

Epithelial-mesenchymal transition (EMT) is an important event in cell development in which epithelial cells acquire mesenchymal fibroblast-like features, including reduced intercellular adhesion and increased motility (91). Among the three subtypes of EMT, type 2 is associated with wound healing, tissue regeneration, and organ fibrosis as a repair-related process. HSP27 is known to increase cell migration and invasion and to mediate EMT in cancer cells, leading to cancer progression. The inhibition of HSP27 is considered a promising therapeutic approach for controlling cancer growth and fibrosis. Oh et al. (92) found that the HSP27 inhibitor J2 increased the lysosomal degradation of HSP27 and promoted STAT6-induced Ym1 expression in M2 macrophages, inhibited IL-8 production, and attenuated inflammatory lung fibrosis in mice. Further studies have revealed that HSP27 activates the NF-κB pathway through direct interaction with IκBα, leading to increased expression of Twist, IL-1β, and IL-6, and promoting the EMT process that is closely associated with the development of radiation-induced pulmonary fibrosis (93). EMT is also an important contributor to the pathogenesis of liver fibrosis. Mangiferin prevented CCl_4_ and TGF-β1-induced EMT and hepatic fibrosis by reducing HSP27 expression to inhibit the JAK2/STAT3 pathway, which in turn inhibited TGF-β1/Smad pathway activation (94). It was found that HSP27 interacted directly with small ubiquitin-related modifier 2/3, regulating pyruvate kinase M2 and promoting the migration and motility through the EMT pathway (95). Studies related to the regulation of EMT by HSP27 have mainly focused on tumor therapy (96, 97), finding that HSP27 is directly involved in EMT in lung mesothelial, epithelial, and colon adenocarcinoma cells. The regulatory role of HSP27 in the fibrosis of other tissues and organs needs to be explored further (Table 1).

**Table 1:** Basic medical research on HSP27.

Pathophysiological mechanisms	Samples	Models/method	Pathway/target	Ref.
Oxidative stress	Human	HCM cardiomyocytes	Administration of HSPs *in vitro*→ oxidative stress↓→impaired PQS↓→HCM cardiomyocyte stiffness↓	(32)
	Mice	PRL and/or cytokine-treated MIN6 beta-cells	G6PD activity ↑ and maintain GSH levels → oxidative stress ↓	(33)
	Mice	MDMA, ethanol or both	Trx-1 in right ventricle↑→ oxidative stress ↓	(34)
	Rats	H_2_O_2_	HSP27 overexpression→ reduced states of GR, Prx1, and Trx 1↑→GSSG↓ and reduced/oxidized GSH ratio↑	(35)
	Rats	H_2_O_2_	HSP27 overexpression→ dephosphorylation of MST1↑→phosphorylation of LATS1 and YAP↓→YAP nuclear localization↑→Hippo tumor-suppressor pathway↓	(35)
	Drosophila melanogasters	Nonylphenol	HSP27 overexpression→ nonylphenol-induced intracellular ROS, LPO, PC content↓, GSH↓	(36)
	Mice	LPS	HSP70 and HSP27↑→soluble SOD1 activity↑→ROS↓	(37)
	Mice	Radiation	HSP27 inhibitor (J2)→radiation-induced ROS production↓→immune cells recruitment↓	(38)
	Human	UVB	HSP27 knockdown→ cell viability↓ and incidence of UVB-induced ROS production↑	(39)
	Mice	UVB	HSP27 knockdown→p62↑, LC3B, CAT, SOD1 and SOD2↓→ROS↑	(39)
	Human	DSP induce HSkMC impairments	IPIRM→CD36, HSP27 and FABP4↓→SOD activity↑, MDA content and CK activity↓	(40)
	Wood frog rana sylvaticas	Freezing stress	Freezing stress→p38 kinase activity↑→HSP27 phosphorylation↑→cytoplasmic SOD1↑	(41)
	Rats	Ischemia-reperfusion injury	ATM kinase→HSP27 phosphorylation at serine 85↑→G6PD activity in pentose phosphate pathway↑→NADPH/NAD+ ratio↑	(42)
	Rats	Ischemia-reperfusion injury	Geranylgeranylacetone→ phosphorylation of HSP27↑→G6PD activity↑→PC↓	(43)
Inflammatory	Human	Chronic HF patients	The combination of sST2, HSP27 and hsCRP is an independent predictor of cardiovascular death and unplanned HF associated hospitalization.	(45)
	Human	Patients with aggressive periodontitis	Systemic HSP27 levels correlated with pro-inflammatory cytokines TNF-α and IL-6	(46)
	Human	TcdB	Overexpressing HSP27→binding of CFTR to NHERF1 and NHERF2↓→decrease of trans-epithelial resistance↓→intestinal barrier function↑	(47)
	Piglets	E. coli supernatant, thallus and LPS	Overexpressing HSP27→adhesion ability of E. coli to IPEC-J2 and IL-1β, IL-6, TNF-α↓; Overexpressing HSP27→*RSK2* in the MAPK pathway↑	(48)
	Mice	Palmitate-induced C2C12 myocytes and HFD-induced obese animal models	Valdecoxib→ AMPK phosphorylation and HSP27↑→NF-κB and IκB phosphorylation, eIF2α phosphorylation, CHOP↓→TNF-α and IL-6↓→inflammatory response↓	(49)
	Rats	Renal ischemia-reperfusion injury	HSP70, HO-1, and HSP27↑→IRI induced lipid peroxidation, inflammation, and apoptosis↓?	(50)
	Mice	MI	Leucocyte infiltration↑, inflammatory cytokines↑, and TLR4/MyD88/NF-κB activation↑	(51)
	Mice	Hypoxic	NF-κB↑→proinflammatory mediators↑→leucocyte recruitment↑→inflammation↑	(51)
	Human	Exposing to UV light induced DNA damage	HSP27 knockdown→E2F-4/p130↑→G2/M-related genes (CCNA2, CCNB1, CCNB2, CDC25C, CDCA3, and CDK1) ↓→G2 arrest→ inflammatory cytokines↑ and cellular senescence	(52)
	Human	Thrombin	GPCR-induced p38-dependent MK2-MK3 inflammatory signaling pathway↑→HSP27 phosphorylation and activation→ endothelial barrier recovery↑	(53)
	Mice	Allergen-induced inflammation in transgenic mice	Noxa required the interaction with phosphorylated HSP27→degradation of ubiquitylated IκBα↓→nuclear translocation and activation of NF-κB↓→allergen-induced inflammation↓	(54)
	Human	LPS	Early inflammation: Phosphorylated HSP27→ p53 activation→ CBP↑→ NF-κB activation↑→release of TNF-α, IL-6, IFN-β, and IL-10↑; Late stages of inflammation: Phosphorylated HSP27→ROS↓→CBP↓	(55)
	Human	LPS	HSP27 phosphorylation↓→phosphorylation of IκBα and activation of NF-κB↓→apoptosis, growth inhibition, and inflammatory factor expression↑	(56)
	Human	LPS	HSP27 phosphorylation→HSP27 bound to CBP→CBP acetyltransferase activity and CBP-dependent acetylation of HMGB1↓→HMGB1 cytosolic translocation↓→inflammatory↓	(57)
	Human	THP-1 MΦ	HSP27 IC interacts with TLR4→NF-κB pathway↑→IL-10↑ and IL-1β↓; HSP27 IC competes with oxLDL→ binding to scavenger receptors SR-AI and CD-36→oxLDL internalization and foam cell formation↓	(59)
	Human	THP-1 MΦ	HSP27 IC→ exosomal abundance and secretion of cholesterol contents↑	(60)
	Human	THP-1 MΦ	HSP27-laden exosomes→ NF-κB activation and release of IL-10↑	(62)
Apoptosis	Mice	ApoE−/− mice	HSP27 knockdown→ Bax, caspase-3↑, Bcl-2↓ in AS plaque→ mitochondria apoptosis pathway↑	(63)
	Mice	Ischemia/reperfusion injury	MicroRNA-410→HMGB1↓→HSP27 activity↓→mitophagy↓→cardiomyocyte injury↓	(64)
	Rats	Lumbosacral nerve root avulsion	Up-regulation of HSP27→apoptosis↓	(65)
	Human	Oxygen-glucose deprivation	Up-regulation of HSP27→GSH content and SOD activity↑→oxidative stress↓; HSP27 knockdown→p65 phosphorylation↓→ NF-κB activation↓	(65)
	Rats	SAH model	Overexpression HSP27→ SAH-elevated activation of MKK4, JNK, c-Jun, and caspase-3↓	(66)
	Rats	UVB	Down-regulating HSP27→MDA↑, HYP↓, Bax↑, cell senescence associated proteins (p16, p53, and p21) ↑→apoptosis↑	(68)
	Human	UVB	HSP27→phosphorylated-Akt-dependent pathway↑, p53/Bax/Bcl-2-dependent mitochondrial apoptotic pathway↓→cytoplasmic localization of p21↑	(69)
	Human	PRAs	HSP27/PI3K/AKT pathway↑→caspase-3 activity↓→ER stress-triggered apoptosis↓	(70)
	Rats	Intravitreal HSP27 injection	Caspases-3, 8, and 9↑	(71)
	Human	Resveratrol	Resveratrol + HSP27-specific siRNA→caspase-3-dependent apoptosis↑	(72)
	Human	Human colon cancer cell line SW480	Suppression of HSP27→NOTCH1↓, Akt and mTOR phosphorylation↓→apoptosis induced by Akt-mTOR signaling pathway↑	(73)
	Human	Human colon cancer cell line HT-29 and DLD-1	Silencing of HSP27→p-Akt/Akt→ ROS/RNS, superoxide, and autophagy↓→apoptosis and reactive oxidative stress production↓	(74)
	Human	x-ray radiation	MiR-541-3p targeting 3′-UTR of HSP27 mRNA→HSP27↓→β-catenin↓→apoptosis post radiation↑	(75)
	Human	Lung epithelial cell transformation under heat stress	Heat stress→miR-541 accumulation↓→HSP27↑→autophagy↑, mitochondrial apoptotic pathway↓	(76)
	Human	NSCLC cells induced by irradiation	Silencing of HSP27→cell viability↓, depolarizing the MMP, arresting the cell cycle in the G2/M phase and cell apoptosis↑	(77)
	Mice	Chemotherapy-induced peripheral neuropathy	Overexpression of HSP27→inactivating caspase-3 activity, RhoA and its downstream effectors inactivation → anti-apoptotic and maintained axon integrity	(78)
	Mice	Chemotherapy-induced peripheral neuropathy	Overexpression of HSP27→axonal degeneration, demyelination, mitochondrial dysfunction, and apoptosis↓	(79)
	Mice	Dox-induced dilated cardiomyopathy	Phosphorylated HSP27→p53-dependent apoptosis↓	(80)
	Human	TNF-α	Glucocorticoid→TNF-R2↑→p38MAPK-HSP27↓→HSP27 phosphorylation↓→apoptosis↓	(82)
	Human	High-glucose	Visfatin→PI3K/Akt and ERK1/2↑→HSP27 phosphorylation↑→apoptosis↓	(83)
	Human	TⅡA treatment human gastric cell line AGS	TⅡA→HSP27 phosphorylation↑→ROS production and unfolded protein response↑→HSF-1 and downstream targets of HSF-1↑→apoptosis↑	(84)
	Human	PDAC cells following exposure to cytotoxic chemotherapy	FIRINOX→TNFR1↑→apoptotic signaling and TAK1-p38-MK2-HSP27 survival pathway involved in treatment resistance↑	(85)
	Human	Lung SCC cells treated with dioscin	Dioscin→p38MAPK/HSP27↑→ROS-induced apoptosis↑	(86)
Fibrosis	Mice	Thrombopoietin- and JAKV617F-induced myelofibrosis	HSP27 regulates the proliferation of JAK2V617F-positive cells and interacts directly with JAK2/STAT5.	(88)
	Human and mice	TGF-β treated HK-11 cells and in kidneys of diabetic mice	HSP27 phosphorylated on Ser82→association of NF-E2/pSer82HSP27↑→targeting NF-E2 to the proteasome→NF-E2 anti-fibrotic action↓	(89)
	Rats	Ischemia/reperfusion in heart of MI rats	Selective delivery of HSP27 to cardiomyocytes → area of fibrosis↓	(90)
	Mice	Irradiation or bleomycin-induced pulmonary fibrosis	HSP27 inhibitor J2→lysosomal degradation of HSP27↑ and STAT6-induced Ym1 expression in M2 macrophages↑→IL-8↓→inflammatory pulmonary fibrosis↓	(92)
	Mice	Radiation	Direct interaction between HSP27 and IkBα→NF-κB↑→Twist, IL-1β, and IL-6↑→radiation-mediated EMT↑	(93)
	Rats and human	CCl_4_- and TGF-β1-induced liver fibrosis and hepatocyte EMT	Mangiferin→HSP27↓→JAK2/STAT3 pathway↓→TGF-β1/Smad signaling↓	(94)
	Human	Esophageal squamous cell carcinoma	HSP27 directly interacted with SUMO2/3→regulates PKM2→infiltration and metastasis through the EMT pathway↑	(95)

AKT, protein kinase B; AS, atherosclerosis; ATM, ataxia telangiectasia mutated; CAT, catalase; CBP, CREB-binding protein; CFTR, cystic fibrosis transmembrane regulator; CHOP, C/EBP homologous protein; CK, creatine kinase; Dox, doxorubicin; DSP, dexamethasone sodium phosphate; eIF2α, eukaryotic initiation factor 2 alpha; EMT, epithelial-mesenchymal transition; GPCR, G-protein-coupled receptors; GR, glutathione reductase; GSH, glutathione; GSSG, glutathione disulfide; G6PD, glucose-6-phosphate dehydrogenase; HCM, hypertrophic cardiomyopathy; HF, heart failure; HMGB1, high mobility group box-1 protein; hsCRP, high sensitivity C-reactive protein; HSF1, heat shock transcription factor 1; HSkMC, human skeletal muscle cell line; HSP, heat shock proteins; HSP27, heat shock protein 27; HUVEC, human umbilical vein endothelial cells; HYP, hydroxyproline; IC, immune complex; IL-6, interleukin 6; IPIRM, intermittent pressure imitating rolling manipulation; JNK, c-Jun N-terminal kinase; LPO, lipid peroxidation; LPS, lipopolysaccharide; MAPK, mitogen-activated protein kinases; MDA, malondialdehyde; MDMA, methylenedioxymethamphetamine; MI, myocardial infarction; MKK4, MAP kinase kinase 4; MST1, mammalian STE20-like kinases 1; MyD88, myeloid differentiation factor 88; NF-κB, nuclear factor-κB; NHERF1, Na^+^/H^+^ exchange regulatory factor-1; oxLDL, oxidized low-density lipoprotein; PC, protein carbonyl; PDAC, pancreatic ductal adenocarcinoma; PI3K, phosphatidylinositol 3-kinase; PKM2, pyruvate kinase M2; PQS, protein quality control system; PRAs, penicillinase-resistant antibiotics; Prx1, peroxiredoxins-1; ROS, reactive oxygen species; RSK2, Ribosomal S6 kinase; SAH, subarachnoid hemorrhage; SOD, superoxide dismutase; sST2, soluble suppression of tumorigenicity-2; SUMO2/3, small ubiquitin-related modifier 2/3; TcdB, toxin CD B; TLR4, toll-like receptor 4; TNF-α, tumour necrosis factor alpha; TNF-R2, tumor necrosis factor receptor-2; Trx-1, thioredoxin-1; UVB, ultraviolet rays B; YAP, Yes-associated protein.

## 4. The therapeutic intervention of HSP27

### 4.1. Atherosclerosis

HSP27 is a traditional intracellular chaperone protein, but it also functions as an extracellular signal that regulates lipid accumulation and foam cell formation, contributing to the prevention of atherosclerosis (98). Clinical studies found that patients with atherosclerosis with >50% coronary stenosis had lower levels of HSP27 than those without atherosclerosis. Follow-up found that low HSP27 levels were associated with the presence of coronary artery disease and the occurrence of future adverse clinical events (99). In a study of 852 patients with stroke and coronary atherosclerosis (100), vitamin D was found to improve oxidative stress by reducing serum anti-HSP27 antibody levels, which may reduce CVD risk; however, further evaluation in larger populations is needed. Circulating HSP27 was positively correlated with carotid intima-media thickness, an independent predictor of early diabetic atherosclerotic lesions, and may represent a novel marker of subclinical atherosclerosis in type 2 diabetes (101). Serum HSP27 and its phosphorylation levels were lower in patients with lower extremity occlusive atherosclerosis and negatively correlated with disease severity (102). Low serum HSP27 levels were independently associated with the occurrence of CVD and sudden cardiac death in patients undergoing dialysis as well as with carotid atherosclerosis and oxidative stress. HSP27 may affect oxLDL-stimulated atherosclerosis by competing with oxLDL for entry into macrophages, reducing ROS production in endothelial cells, and reducing oxidative modifications of LDL (103).

HSP27 exerts a potential therapeutic effect on atherosclerosis by improving endothelial function. Intermittent hypoxia exposure (IHE) can increase the production of ROS and erythropoietin, which is adapted to strenuous exercise. Several studies have demonstrated the protective effects of moderate hypoxia on CVD. Researchers have demonstrated for the first time that IHE combined with physical activity can reduce endothelial dysfunction and atherosclerosis risk by increasing nitric oxide bioavailability and circulating HSP27 levels (104). However, further research is needed to translate hypoxic exposure interventions to the clinical setting. In a study of HSP27-deficient and overexpressing mice, Venu et al. (105) found that HSP27 can affect mRNA levels of endothelial nitric oxide synthase (eNOS) and soluble guanylate cyclase, as well as the proportion of vasodilation that is sensitive to the action of L-N^G^-nitro arginine methyl ester (L-NAME), thereby influencing nitric oxide-mediated vascular relaxation by regulating intact vascular endothelial function.

Additionally, HSP27 can improve atherosclerosis through anti-inflammatory, anti-stress, and anti-aging pathways. Heat treatment can improve atherosclerotic lesions by upregulating the expression of aortic SIRT1, HSF1, HSP27, HSP72, and HSP73, significantly reducing the plasma levels of triacylglycerol, total cholesterol, and LDL cholesterol, and restoring anti-inflammatory and anti-aging HSRs (106). 4-Phenyl butyric acid (4-PBA) is a small chemical molecular chaperone that was shown to inhibit the growth of atherosclerotic plaques by increasing the expression of HSP27 in ApoE−/− mice. *In vitro* experiments indicated that 4-PBA treatment inhibited macrophage attachment to human aortic endothelial cells, prevented ER stress-induced cell death, increased the nuclear localization of HSF1 and the expression of HSP27, and may have exerted a therapeutic effect on atherosclerosis through this pathway; however, the exact mechanism needs to be investigated further (107).

In recent years, immunotherapy has become a popular topic in the field of oncology. Next, we review immunotherapy with HSP27 for atherosclerosis that may have efficacy. Previous studies have confirmed that HSP27 is an estrogen receptor β-associated protein and that estrogen induces elevated levels of HSP27 and anti-HSP27 antibodies in healthy subjects compared to the levels in patients with CVD. Experiments on ovariectomized ApoE−/− mice to mimic human menopause found a 65% increase in atherosclerosis burden compared to that in the sham-operated group (28). However, after administration of recombinant HSP27 (rHSP27), atherosclerotic lesions significantly improved. In-depth studies have revealed that the IC formed by rHSP27 vaccination upregulated hepatocyte low-density lipoprotein receptor (LDLR) expression via the NF-κB pathway, leading to increased LDLR for binding proprotein convertase subtilisin/kexin type 9 (PCSK9) and mediating its lysosomal processing (108). Thus, HSP27 immunotherapy is a promising therapeutic strategy. It lowers cholesterol levels by upregulating LDLR expression, promotes clearance of LDL cholesterol, and reduces atherosclerosis formation; however, in-depth studies for clinical applications are warranted.

### 4.2. Ischemic heart disease

Numerous studies have demonstrated the protective role of HSP27 in reducing ischemic oxidative stress, its high level of endogenous expression in human myocardial tissue, and its apparently important role in improving the development of ischemic heart disease (IHD). Clinical studies in large cohorts have noted that serum HSP27 concentrations are elevated in the first few hours after acute coronary syndrome but fall to levels close to those of healthy individuals approximately 12 h after the onset of chest pain (109). The level of HSP27 expression in peripheral blood mononuclear cells of patients with IHD correlated significantly with disease severity in patients with ≥50% coronary stenosis and can be used as an early prognostic biomarker (110). A study of 400 patients with IHD (111) found that anti-HSP27 titers were significantly higher in patients with IHD than in the control patients. In addition, within the IHD group, anti-HSP27 titers were higher in patients with three-vessel disease than in those with two-vessel and one-vessel disease. Serum anti-HSP27 titers may be associated with the presence and severity of coronary artery disease.

Platelet hyperactivity is a well-known risk factor for IHD and thrombosis. The function of HSP27 is regulated by phosphorylation, which may reflect its cytoprotective function; however, the precise mechanism is not fully understood. Compared with patients with non-ischemic chest pain, patients with MI have significantly increased platelet HSP27 levels and phosphorylation, accompanied by characteristic intracellular translocation of HSP27 from the cytoskeleton to the platelet membrane (112). The future extension of this platelet HSP27 phenotype to other acute ischemic events would be interesting. Chemokine (C-C motif) ligand 2 (CCL2) promotes inflammatory responses and accelerates the progression of IHD. CCL2 knockout mice exhibited markedly diminished platelet aggregation and secretion, accompanied by reduced phosphorylation of protein kinase C alpha (PKCα), p38 MAPK, and HSP27 (113). In-depth studies have revealed that CCL2 increases platelet aggregation, activation, and granule secretion by triggering the PKCα/p38 MAPK/HSP27 signaling pathway in platelets, regulating platelet function, and thus affecting arterial thrombosis. The tomato extract Fruitflow® restored collagen-activated platelet cyclic AMP levels, reduced collagen-induced phosphorylation of protein kinase A substrates, and reduced platelet AKT, glycogen synthase kinase 3β, p38 MAPK, and HSP27 phosphorylation, which is beneficial in people at risk of platelet hyperactivity-induced thrombosis (114). Altered ion homeostasis during I/R is an important trigger of cell death. Inhibition of electrogenic sodium bicarbonate co-transporter isoform 1 (NBCe1) can increase HSP27 phosphorylation while attenuating Drp1-dependent mitochondrial fission by attenuating calcium overload and activating the calcium-regulated neurophosphatase/p38 MAPK/HSP27-dependent pathway, which can improve mitochondrial status and cardiac function after myocardial ischemia (115). Adenine nucleotide translocase (ANT) plays a central role in cellular energy supply and mutations in *ANT1* in the heart have been associated with IHD (116). In a rat MI model and a primary cardiomyocyte hypoxia model (117), cardiomyocytes overexpressing ANT1 had enhanced HSP27 expression and secretion under hypoxic conditions; the secreted HSP27 induced the expression of HSP27 and ANT1 by stimulating TLR4-dependent AKT activation. Increased levels of ANT1 and HSP27 expression increased the stability of the mitochondrial membrane potential and inhibited caspase-3/7 activity, exerting cardioprotective effects.

### 4.3. Atrial fibrillation

Atrial fibrillation (AF) is the most common arrhythmia worldwide and is associated with high morbidity and mortality from ischemic stroke and HF. Disruption of protein homeostasis is the basis of structural and electrical conduction damage. Cells respond to loss of proteostasis by inducing HSR, leading to HSP expression. The sHSP family, which includes HSP27, may be important in maintaining cardiomyocyte proteostasis by stabilizing contractile proteins and may serve as a potential biomarker for predicting AF recurrence after treatment (118).

In a study of 114 patients with AF (119), higher baseline HSP27 levels after catheter ablation were found to predict sinus rhythm maintenance in patients with paroxysmal AF. Baseline HSP27 levels also correlated with IL-10 and TNF-α levels, suggesting that the predictive value of HSP27 in postoperative AF patients may be related to inflammatory responses. A study of 300 patients with AF (120) found that serum HSP27 levels were significantly increased at 3, 6, and 12 months after ablation in patients with AF recurrence compared to those in patients without AF recurrence within 1 year after ablation. Serum HSP27 levels were significantly elevated in patients with AF recurrence within one year of pulmonary vein isolation, and HSP27 levels were predictive of AF recurrence after ablation therapy. In patients with rheumatic heart disease and AF, HSP27 levels were negatively correlated with AF duration and left atrial diameter. Left atrial enlargement and low HSP27 expression were independent predictors of AF in patients with rheumatic heart disease (121).

By establishing an experimental model of chronic AF in dogs, it was found (122) that atrial HSP27 mRNA and protein expression levels were significantly higher in the paced group than in the sham-operated group, while those in the paced + angiotensin 1–7 (Ang-(1–7)) group were significantly lower than those in the paced group. These findings suggest that overexpression of HSP27 is an atrial tissue response to rapid atrial pacing, and Ang-(1–7) may indirectly downregulate the expression of HSP27 by improving atrial remodeling. Previous studies have revealed that HSP-inducing compounds, such as L-glutamine, can reduce the onset and progression of AF. L-glutamine supplementation reduced the serum levels of HSP27 and HSP70 over 3 months and normalized the levels of several metabolites associated with carbohydrate, nucleotide, amino acid, vitamin, and cofactor metabolic pathways (123). Future in-depth investigations of the regulatory role of L-glutamine in AF may be beneficial for its application in clinically therapeutic areas.

### 4.4. Cardiomyopathy

Cardiomyopathies include a heterogeneous group of diseases characterized by mechanical or electrical disturbances of the myocardium. The etiology of cardiomyopathy is diverse, with the end result being ventricular dysfunction and progressive HF. HSP27 has potential therapeutic value in myocardial diseases with multiple etiologies. Reduced myocardial diastolic function is an early manifestation of diabetic cardiomyopathy. Studies in diabetic rats found that aerobic exercise increased the left ventricular end-diastolic internal diameter and left ventricular end-diastolic volume, upregulated myocardial HSP27 expression, increased HSP27 phosphorylation at the Ser82 site, and enhanced myocardial pHSP27-myosin co-localization in diabetic rats, suggesting that exercise reduces cardiac diastolic dysfunction and repairs damaged myocardial proteins in diabetes (124). Cardiomyocytes from patients with HCM exhibited higher passive stiffness (F_passive_). Protein kinase D treatment increased HSP27 phosphorylation and restored intracellular localization of HSP27 to the Z-disc and I-band, decreasing F_passive_ and producing a therapeutic effect on the diastolic left ventricular dysfunction associated with high cardiomyocyte stiffness (125). The term “desminopathies” refer to a clinically heterogeneous group of familial and sporadic myopathies and cardiomyopathies that are caused by mutations in the human desmin gene on chromosome 2q35 (126). Studies using an R349P desmin knock-in mouse model revealed that the mutant desmin increased proteasome activity, stimulated macroautophagy, induced chaperone-assisted selective autophagy dysregulation, and increased protein levels of αB-crystallin and HSP27, which translocated from the Z-disc to the level of I-band myelin. This finding provides a basis for further pharmacological and genetic intervention studies (127) (Table 2).

**Table 2:** Applications of HSP27 in cardiovascular diseases.

Disease	Research object	Pathophysiological mechanisms	Ref.
Atherosclerosis	Patients with >50% stenosis in any major epicardial artery	Low serum HSP27 levels are associated with the presence of coronary artery disease and prognostic of future adverse clinical events.	(99)
	Patients with stroke and heart atherosclerotic disorders	Serum vitamin D→anti-HSP27 antibody titers↓→oxidative stress↓→risk of CVD↓	(100)
	Individuals with type 2 diabetes	Circulating HSP27, positively correlates with carotid IMT, is an independent predictor for early atherosclerotic changes in diabetes.	(101)
	Patients with lower extremity arteriosclerosis obliterans	HSP27 and its phosphorylation of lesion group↓	(102)
	Hemodialysis patients	Low serum HSP27 level is independently associated with both CVD, carotid atherosclerosis and oxidative stress.	(103)
	Intermittent hypoxic exposure combined with physical activity in athletes	NO bioavailability and circulating HSP27 level↑→endothelial dysfunction↓	(104)
	HSP27-null and HSP27 overexpressing mice	HSP27↑→levels of mRNA for eNOS and soluble guanylyl cyclase and the proportion of vasorelaxation that is sensitive to the action of L-NAME↑→endothelial function↑	(105)
	Mice fed a high-fat diet	Heat treatment→ aortic expressions of SIRT1, HSF1, HSP27, HSP72 and HSP73↑→TG, total cholesterol, LDL-cholesterol↓→anti-inflammatory and anti-senescent heat shock response↑	(106)
	4-PBA–treated ApoE−/− mice	4-PBA→HSP27↑→atherosclerotic lesion growth↓	(107)
	4-PBA–treated human monocyte/macrophage (THP-1) cells	4-PBA→nuclear localization of HSF-1↑ →HSP27↑	(107)
	ApoE−/− mice subjected to ovariectomy after a high fat diet	Recombinant HSP27 vaccination →atherogenesis and cholesterol levels↓	(28)
	ApoE−/− mice fed a high fat diet and vaccinated with recombinant HSP27	HSP27 immune complex→ NF-κB↑→LDLR↑→PCSK9↓→inflammation↓	(108)
Ischemic heart disease	Patients with acute coronary syndrome	Serum HSP27 in the early hours↑	(109)
	IHD patients	The increased expression of HSP27 in PBMCs of IHD patients is significantly correlated with IHD severity in patients having ≥50% stenosis.	(110)
	IHD patients	Serum anti-HSP27 titers may be associated with the presence and severity of coronary artery disease.	(111)
	STEMI patients	HSP27 protein levels and phosphorylation of HSP27 in human platelets↑ and characteristic intracellular translocation of HSP27 from the cytoskeletal into the membrane.	(112)
	STEMI patients and CCL2−/− mice	CCL2→PKCα/P38MAPK/HSP27 signaling in platelets↑→platelet aggregation, activation and granule secretion↑	(113)
	Human platelet aggregation induced by collagen	Fruitflow® recovered cyclic adenosine monophosphate levels→ protein kinase A substrate phosphorylation↓→Akt, glycogen synthase kinase 3β, p38MAPK, and HSP27 phosphorylation↓	(114)
	Isolated rat hearts with ischemia/reperfusion	NBCe1↓→Ca^2+^ overload↓→calcineurin/p38MAPK/HSP27↑→HSP27 phosphorylation↑→myocardial post-ischemic contractility↑	(115)
	ANT1 transgenic cardiomyocytes under hypoxic conditions and MI rats	ANT1-overexpressing cardiomyocytes under hypoxic conditions→HSP27↑→TLR4-dependent AKT activation↑→HSP27 and ANT1↑→caspase-3/7↓→cellular protection↑	(117)
Atrial fibrillation	Patients with AF	After catheter ablation, a high baseline HSP27 level could predict sinus rhythm maintenance in the patients with paroxysmal AF.	(119)
	Patients with symptomatic AF	HSP27 levels were increased in serum samples of patients with AF recurrence within one year after PVI.	(120)
	Rheumatic heart disease patients with AF	HSP27 levels were negatively associated with AF duration and left atrial diameter.	(121)
	Canine hearts with rapid atrial pacing	Atrial HSP27 mRNA and protein for the pacing group↑	(122)
	AF patients	HSP-inducing compounds L-glutamine→HSP27 and HSP70↓,normalizes metabolite levels→ AF episodes↓?	(123)
Cardiomyopathies	Diabetic cardiomyopathy rats	Aerobic exercise→myocardial HSP27, HSP27-S82 phosphorylation, pHSP27-titin binding↑→left ventricular end-diastolic internal diameter, left ventricular end-diastolic volume↑	(124)
	Hypertrophic cardiomyopathy patients and cardiomyocyte-specific protein kinase D knock-out mice	Protein kinase D→HSP27 phosphorylation↑ and restore HSP27 localization to the Z-disk and I band	(125)
	R349P desmin knock-in myoblasts in conjunction with the corresponding desminopathy mice	Mutant desmin→ proteasomal activity↑, macroautophagy↑, dysregulates the chaperone assisted selective autophagy↑, αB-crystallin and HSP27↑	(127)

AF, atrial fibrillation; ANT, adenine nucleotide translocase; CCL2, chemokine CC-motif ligand 2; CVD, cardiovascular diseases; HSP27, heat shock protein 27; IHD, ischemic heart disease; IMT, carotid intima-media thickness; LDLR, low density lipoprotein receptor; NBCe1, Na^+^/bicarbonate cotransporter e1; PBMC, peripheral blood mononuclear cell; PKCα, protein kinase C alpha; PVI, pulmonary vein isolation; STEMI, ST-segment elevation myocardial infarction; TG, triglyceride; 4-PBA, 4-phenyl butyric acid.

## 5. Prospects and outlook

As an intracellular chaperone protein, HSP27 can be upregulated under a variety of stressful stimuli and mediates pathophysiological processes, such as cellular oxidative stress, inflammatory responses, apoptosis, autophagy, and fibrosis. Numerous studies have revealed its cytoprotective role; however, there are still some questions that need to be addressed, including: Are intracellular and extracellular HSP27 functions consistent? Is the presence of other proteins necessary for HSP27 to perform its physiological function? What is the effect of post-translational modifications other than phosphorylation on HSP27 function? The resolution of these issues is necessary to deeply explore the physiological functions of HSP27 and thus develop disease therapeutics to target it.

In addition, HSP27 can influence the developmental processes of various diseases. HSP27 is strongly induced by physiological stress and anticancer drugs. The powerful cytoprotective function of HSP27 and the fact that this protein is overexpressed in most cancers make this chaperone an attractive target in cancer therapy. HSP27 depletion in various cancer models induces tumor regression, and the antisense oligonucleotide OGX-427, a specific inhibitor of HSP27, is currently undergoing phase II clinical trials. However, its application in the field of cardiovascular disease treatment is still lacking, and therapeutic protocols for improving diseases including atherosclerosis, ischemic heart disease, cardiac arrhythmias, and cardiomyopathies are still at the stage of cell or animal experiments, and the results of relevant clinical studies are needed. Thus, future HSP27-related research in cardiovascular diseases remains to be conducted.
